# Association between continuity of care and detection of hypertension in Dutch general practice: a 10-year cohort study

**DOI:** 10.1136/bmjopen-2025-113374

**Published:** 2026-03-23

**Authors:** Nick Ryan van der Velde, Marije T te Winkel, Jos P Kanning, Birgit I Lissenberg-Witte, Ralf Harskamp, Otto R Maarsingh

**Affiliations:** 1 Department of General Practice, Amsterdam UMC Location VUmc, Amsterdam, Netherlands; 2 Amsterdam Public Health Research Institute, Amsterdam, Netherlands; 3 Julius Center for Health Sciences and Primary Care, Utrecht, Netherlands; 4 University Medical Centre Utrecht, Utrecht, Netherlands; 5 Utrecht University, Utrecht, Netherlands; 6 Department of General Practice, Amsterdam UMC, Amsterdam, Netherlands; 7 Amsterdam Cardiovascular Sciences, Amsterdam, Netherlands

**Keywords:** Primary Care, General Practice, Hypertension, PREVENTIVE MEDICINE, Cardiovascular Disease

## Abstract

**Abstract:**

**Objectives:**

Hypertension is a major modifiable risk factor for cardiovascular disease, and timely detection enables interventions that can substantially reduce this risk. General practice, with continuity of care (COC) as one of its core values, plays a pivotal role in hypertension detection. This study aimed to investigate the association between COC and the detection of hypertension in general practice.

**Design:**

Longitudinal dynamic cohort study.

**Setting:**

This study used routine care data from 48 Dutch general practices between 2013 and 2022.

**Participants:**

106 755 adults without known cardiovascular diseases or risk factors at baseline.

**Outcome measures:**

The Herfindahl-Hirschman Index, an established measure for COC, was used to calculate both general practitioner (GP)- and team-COC. A multivariable Cox proportional hazard regression model was used to assess the association between COC level (low, intermediate, high) and the incidence of hypertension detection.

**Results:**

We included 106 755 patients (59.5% female, median age 35 years) in our analysis. The overall incidence rate was 9.42 hypertension diagnoses per 1000 person-years (95% CI 9.20 to 9.64). Compared with low COC, patients receiving intermediate or high GP-COC had a 1.9 (95% CI 1.7 to 2.1) to 4.9 (95% CI 4.4 to 5.4) higher HR of hypertension detection; patients receiving intermediate or high team-COC had a 2.3 (95% CI 2.2 to 2.5) to 7.3 (95% CI 6.8 to 7.8) higher HR of hypertension detection. High personal continuity was associated with up to 8.3 months (95% CI 8.6 to 7.9) earlier detection of hypertension. The association between COC and hypertension detection was dose-dependent.

**Conclusions:**

This study shows that both GP-COC and team-COC are dose-dependently associated with increased HRs and earlier detection of hypertension in adults without preregistered cardiovascular conditions. Promoting COC contributes to cardiovascular preventive care.

**STRENGTHS AND LIMITATIONS OF THIS STUDY**

- This study is considerably strengthened by calculating both general practitioner-COC and team-continuity of care (COC).
- This study provides quantitative evidence on the association between COC and hypertension detection.
- The relatively large sample size, combined with the 10-year observation period, allows long-term COC monitoring across different disease episodes.
- The generalisability of our findings beyond healthcare systems similar to Dutch primary care remains uncertain.

## Introduction

 Primary hypertension is one of the strongest (modifiable) cardiovascular risk factors (CVRs) for cardiovascular disease (CVD).^1^,^4^ Long-standing elevated blood pressure (BP) and/or hypertension causes organ damage and leads to cardiovascular, cerebrovascular and renal disease.^5^ In 2019, the median age-standardised prevalence of hypertension in European Society of Cardiology (ESC) member countries was 36% (IQR 26%–41%) among women and 41% (IQR 35%–48%) among men.^6^ Globally, only 21% of the estimated 1.28 billion individuals with hypertension receive adequate therapy.^7^ As the global prevalence of CVDs rises, early detection and management of CVRs is increasingly important and is found to be one of the key challenges in cardiovascular research.^8^ In the UK, 90% of all adults >40 years have a BP check within a 5-year time period.^9^ The ESC, however, recommends regular opportunistic BP screening in all adults attending healthcare facilities to enable early detection of hypertension, at least every 3 years for adults <40 years and annual screening for adults ≥40 years.^5^

General practitioners (GPs) have a principal role in detection, monitoring and optimising hypertension and reducing the incidence of and premature mortality due to CVDs.^10 11^ However, general practice faces high workloads with additional administrative and organisational burdens, increasing staff shortages, increasing health costs,^12^ an ageing population^13 14^ and an increasing demand for (complex) care.^15^ This, among an intensifying national shortage of GPs, contributes to a substantial and increasing mismatch between supply and demand and pressure on Dutch GP care.^1215^,^19^ Meanwhile, CVDs and related risk factors are an increasingly important driver of healthcare costs.^20 21^ In the USA, for example, the health costs due to hypertension alone are projected to increase by 220% between 2020 and 2050.^21^ Promoting continuity of care (COC) is emerging as a potential remedy for these challenges. COC encompasses the familiarity and mutual trust established between a patient and their regular healthcare provider(s) through repeated encounters over time^22^ and is recognised as a fundamental principle of general practice.^23^,^27^ Numerous studies have demonstrated COC to be associated with multiple benefits for patients, physicians and health systems,^28 29^ including improved medication adherence,^30^,^37^ better uptake of preventive care,^2930 35 38^,^40^ fewer emergency department visits^29 41 42^ and hospital admissions,^2942^,^45^ reduced mortality rates^46 47^ and reduced health costs.^48^,^50^ Even though hypertension is the most common reason for patients in Dutch general practice to contact their GP, accounting for almost 3% of all GP consultations,^51^ studies assessing the relation between COC and CVRs have predominantly focused on condition management in patients with registered diabetes mellitus type 2, hypertension or hypercholesterolaemia,^5052^,^59^ or patient adherence,^60^,^63^ rather than the detection of diagnoses itself.

We hypothesise that increased COC supports timely hypertension detection, which may contribute to both better cardiovascular health outcomes and relieved pressure on GP care. Therefore, the aim of this study was to analyse the time to hypertension detection across various levels of COC. In addition, COC was calculated separately for GPs (GP-COC) and for the entire general practice team (team-COC, detailed in box 1), to align with the team-based provision of COC.^64^

Box 1 **The Herfindahl-Hirschman Index (HHI)**

$Herfindahl\text{-}Hirschman\;Index=\sum_{i=1}^{p}\frac{n_{i}^{2}}{n^{2}}$

The HHI is calculated by using the fraction of contacts with all selected healthcare providers, where *p* is the total number of different providers, *n* is the total number of contacts and *n_i_* is the number of contacts with provider *i*.

The score ranges from 1/*N* (minimal continuity, a different provider for every contact with the GP’s practice) to 1 (maximal continuity, the same provider for all contacts).

- For GP-COC, all GP consultations were included in the HHI calculations.
- For team-COC, all GP consultations and all vis-à-vis consultations with other healthcare providers (nurses, assistants, etc) were included in the HHI calculations.

COC, continuity of care; GP, general practitioner.

## Methods

### Setting

Fal practices affiliated with the Academic Network of Primary Care (ANHA) at Amsterdam University Medical Centre. These practices were in the area in and around Amsterdam and Haarlem, the Netherlands. In the Netherlands, GPs are the primary point of contact for most health issues; in 2024, over 99% of Dutch citizens had a designated general practice (registration by name).^65^ Patients registered with ANHA-affiliated practices could opt out of data sharing at any time, though this occurred infrequently (~0.85%).

### Study population

The dataset encompasses dynamic routine care data from patients registered between 2013 and 2018 with the same general practice for at least 1 year and had a minimum of five contacts, of which at least two were with a GP. These patients were followed up until 1 January 2023. We excluded patients younger than 18 years and those with preregistered CVDs, related conditions, CVRs, as well as oncological diagnoses on 1 January 2013. These conditions were based on the cardiovascular management guidelines of the Dutch College of General Practitioners (NHG) and are detailed in online supplemental table 1.

### Data sourcing

The administrative data includes pseudonymised patient demographics (age, sex), the type of contact (consultation in GP’s practice, home visits, telephone calls or per email), the healthcare provider (GP, locum GP, or other healthcare professionals—unspecified, including nurses and assistants) and the presented health problems, defined as complaints or diagnosis registered by the GP using the International Classification of Primary Care (ICPC).^66^ The date of hypertension detection was defined as the date on which the first episode of hypertension was recorded. All ICPC records were used, both primary and secondary diagnosis positions, depending on the GP’s reporting. Other conditions were considered present if they were not primarily excluded from the analysis or were recorded after 1 January 2013. Demographic data were recorded by practices at the time of patient registration and are available for all included patients.

The start of follow-up was defined as the later of 1 January 2013 or the registration date. The end of follow-up was defined as the earliest of 1 January 2023, the date of hypertension registration, or the date of deregistration. Deregistration from a general practice occurs when a patient chooses to register with another GP, for example, after moving to a new location or in the event of the patient’s death. Finally, baseline practice characteristics such as practice size, the number of (locum) practitioners, their duration of employment and the number of other healthcare providers between 2013 and 2018 were available.

Additionally, per patient’s four-digit postal code lifestyle factors (eg, tobacco and alcohol use, obesity and physical activity), relative socioeconomic status scores (based on household data concerning relative welfare, highest level of education and recent labour participation), ethnicity (percentage of the population in the postal code who, or whose parents, were born in Africa, South America, Asia (excluding Indonesia and Japan) or Turkey) and air pollution (unweighted average particulate matter concentrations) were added from publicly available sources of the National Institute for Public Health and the Environment (RIVM) and Statistics Netherlands (CBS). For detailed information on the methodology of the publicly available sources, see online supplemental methodology S2.

The Herfindahl-Hirschman Index (HHI) was calculated to assess the level COC (box 1). To measure GP-COC, all GP consultations (consultations at the GP’s practice, home visits, consultations by telephone and e-consultations) were included in the HHI calculations. To measure team-COC, all GP consultations and vis-à-vis consultations with other healthcare providers, including but not limited to regular and locum practice nurses and medical assistants, were also included in the HHI calculations. Telephone contacts with GP assistants and nurses were excluded, as most of these contacts include appointment scheduling and prescription refilling. The HHI measures were divided into tertiles to compare ‘low’, ‘intermediate’ and ‘high’ COC.

### Statistical analysis

The data were pre-processed using IBM SPSS Statistics (V.28.0). Statistical analyses were conducted on the final dataset using R (V.4.2.1, using the haven, survival, survRM2 and frailty packages). First, categorical and normally distributed continuous data were summarised with frequencies and percentages and mean and SD, respectively, whereas non-normally distributed data (Pearson’s second median skewness coefficient ≤−0.04 or ≥0.04) were described with the median and IQR (SPSS).

A Kaplan-Meier survival curve was used to visualise the time to hypertension detection across the different COC levels. The unadjusted restricted mean survival time (RMST) was used to estimate differences in time to hypertension detection across COC levels over a 10-year period.^67^,^69^ A multivariable Cox proportional hazards regression model was used to assess the association between COC levels and hypertension detection, adjusting for clustering at the practice level using a frailty term to account for the fact that individual patients within the same practice may have similar risks due to shared practice characteristics that were not measured.

To assess potential confounding and effect modification, selected covariates were a priori selected using a conceptual approach informed by clinical reasoning and prior literature. Descriptive statistics were examined to support these decisions. First, data were stratified for effect modifiers when the interaction term had a p value <0.001. Data were adjusted for confounding when the regression coefficient changed ≥10%.

Since our study relies on routine care data, which reflect GP-recorded contacts and diagnoses rather than the actual presence of conditions, unrecorded conditions were assumed to be absent.

### Patients and public involvement

This study used existing routine care data from Dutch general practices to investigate the association between COC and hypertension detection. It did not involve patients or the public in the design, conduct, reporting or dissemination of the study findings.

## Results

### Cohort analysis

**Figure 1:**
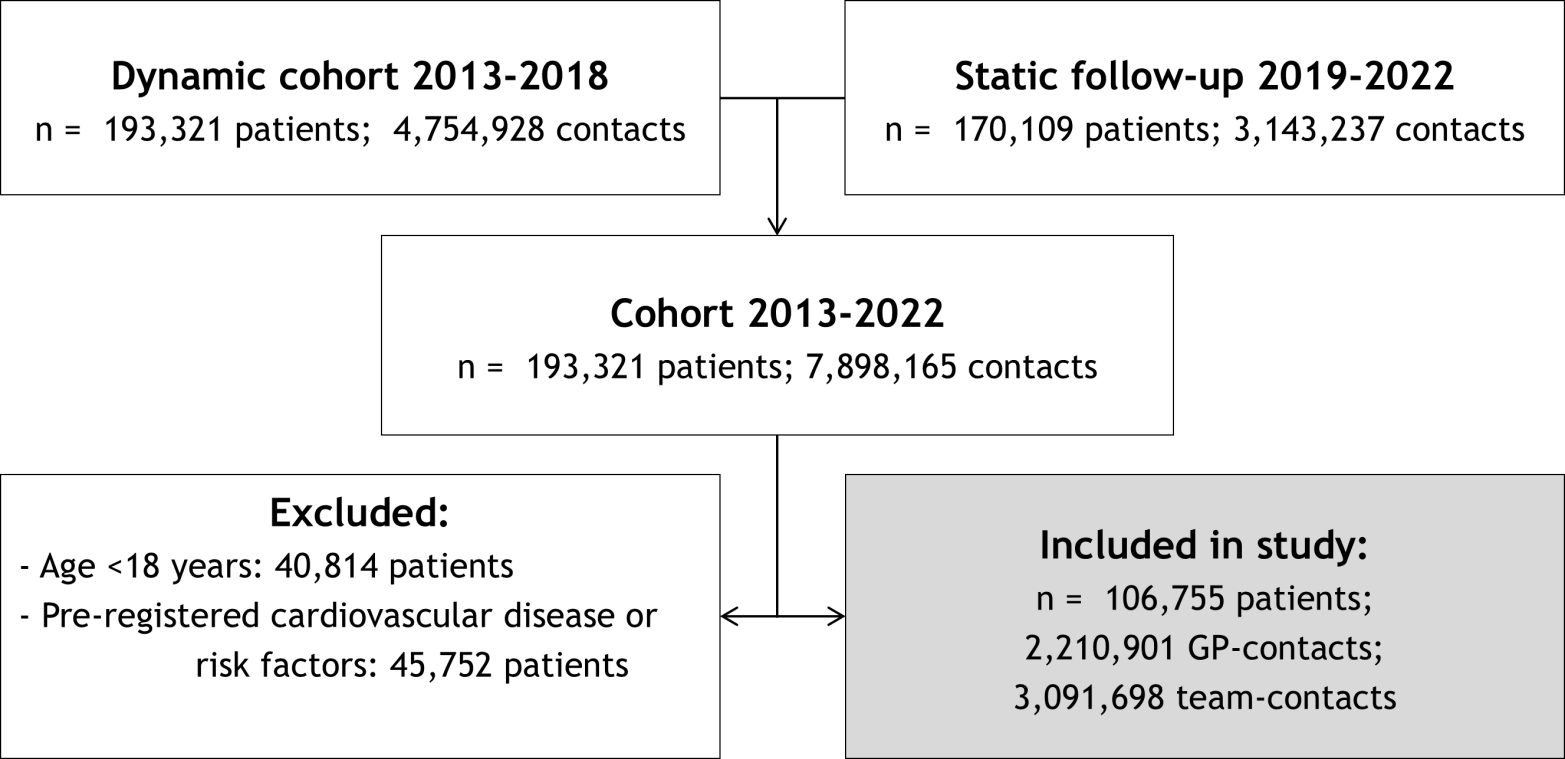
A flowchart illustrating the inclusion and exclusion criteria for patient contacts with the general practice in the cohorts. GP, general practitioner.

We included 1 93 321 patients who had had 7 898 165 contacts with the general practice between 2013 and 2022. The HHI was calculated based on both GP contacts and contacts with the entire team (assistants, nurses, GPs, trainees). 106 755 patients met the cohort criteria. A flowchart depicting these criteria can be found in figure 1. Online supplemental table S3.1 shows the number of consultations per healthcare profession per setting which are used in the HHI calculations of the included patients.

Of our cohort, 59.5% was female and the median age was 35 years old (IQR 27–48 years) on 1 January 2013. Table 1 summarises the descriptive patient characteristics of the study population per tertile team-COC. Online supplemental table S3.2 summarises the remainder practice, behaviour and environmental characteristics of the study population. The cut-offs for the low, intermediate and high HHI tertiles were 0.09–0.37, 0.37–0.55 and 0.55–1.00 for GP-COC and 0.04–0.22, 0.22–0.36 and 0.36–1.00 for team-COC.

**Table 1:** Descriptive characteristics of the study population

	Team-based continuity of care (HHI)			Total
	Low 0.04–0.22	Intermediate 0.22–0.36	High 0.36–1.00	
Patient characteristics				
Female sex (n, %)	22 514 (63.2)	20 937 (59.4)	20 087 (56.1)	63 538 (59.5)
Age (2013), years (median, IQR)	35 (26–47)	35 (26–47)	36 (28–49)	35 (27–48)
<25 years (n, %)	7225 (20.3)	6466 (18.3)	5347 (14.9)	19 038 (17.8)
25–30 years (n, %)	6827 (19.2)	7080 (20.1)	6941 (19.4)	20 848 (19.5)
30–40 years (n, %)	8463 (23.7)	8680 (24.6)	8777 (24.5)	25 920 (24.3)
40–50 years (n, %)	6238 (17.5)	6426 (18.2)	6609 (18.4)	19 273 (18.1)
>50 years (n, %)	6893 (19.3)	6624 (18.8)	8159 (22.8)	21 676 (20.3)
Follow-up time, months (median, IQR)	120 (78–120)	95 (58–120)	68 (33–120)	93 (56–120)
GP contacts/patient* (median, IQR)	17 (10–28)	14 (8–26)	13 (6–25)	15 (8–26)
GP continuity*, HHI (median, IQR)	0.29 (0.23–0.37)	0.44 (0.36–0.54)	0.65 (0.54–0.82)	0.45 (0.33–0.62)
Team contacts/patient* (median, IQR)	27 (17–44)	20 (11–35)	15 (8–30)	21 (11–37)
Number of contacts/month† (median, IQR)	0.29 (0.18–0.46)	0.25 (015–0.42)	0.24 (0.13–0.42)	0.26 (0.16–0.43)
Low frequent attenders (n, %)	9188 (25.9)	12 297 (35.0)	13 818 (39.2)	35 303 (33.3)
Average frequent attenders (n, %)	13 083 (36.9)	11 615 (33.0)	10 574 (30.0)	35 272 (33.3)
High frequent attenders (n, %)	13 211 (37.2)	11 236 (32.0)	10 871 (30.8)	35 318 (33.4)
Team continuity*, HHI (median, IQR)	0.16 (0.13–0.19)	0.28 (0.25–0.31)	0.50 (0.41–0.61)	0.28 (0.19–0.41)
Cardiovascular diseases and risk factors				
Hypertension (n, %)	1279 (3.6)	1813 (5.1)	4012 (11.2)	7104 (6.7)
Hypercholesterolaemia (n, %)	1930 (5.4)	1568 (4.4)	1754 (4.9)	5252 (4.9)
Diabetes mellitus type 2 (n, %)	852 (2.4)	688 (2.0)	740 (2.1)	2280 (2.1)
Cardiac arrhythmia (n, %)	1111 (3.1)	1049 (3.0)	1232 (3.4)	3392 (3.2)
Congestive heart failure (n, %)	183 (0.5)	228 (0.6)	325 (0.9)	736 (0.7)
Chronic kidney disease (n, %)	700 (2.0)	685 (1.9)	930 (2.6)	2315 (2.2)
Number of chronic diseases‡				
0–2 (n, %)	32 335 (90.7)	32 173 (91.2)	32 137 (89.7)	96 645 (90.5)
3–4 (n, %)	2666 (7.5)	2466 (7.0)	2814 (7.9)	7946 (7.4)
≥5 (n, %)	645 (1.8)	637 (1.8)	882 (2.5)	2164 (2.0)

Additional descriptive characteristics on practices, patients’ behaviour and environmental factors are listed in online supplemental table 3.2.

* Per patient, during follow-up period.

† Sum of contacts with GPs practice during the observation period divided by the total observation period in months.

‡ International Classification of Primary Care’s of chronic diseases are listed in online supplemental table 1.

GP, general practitioner; HHI, Herfindahl-Hirschman Index; n, number.

**Figure 2:**
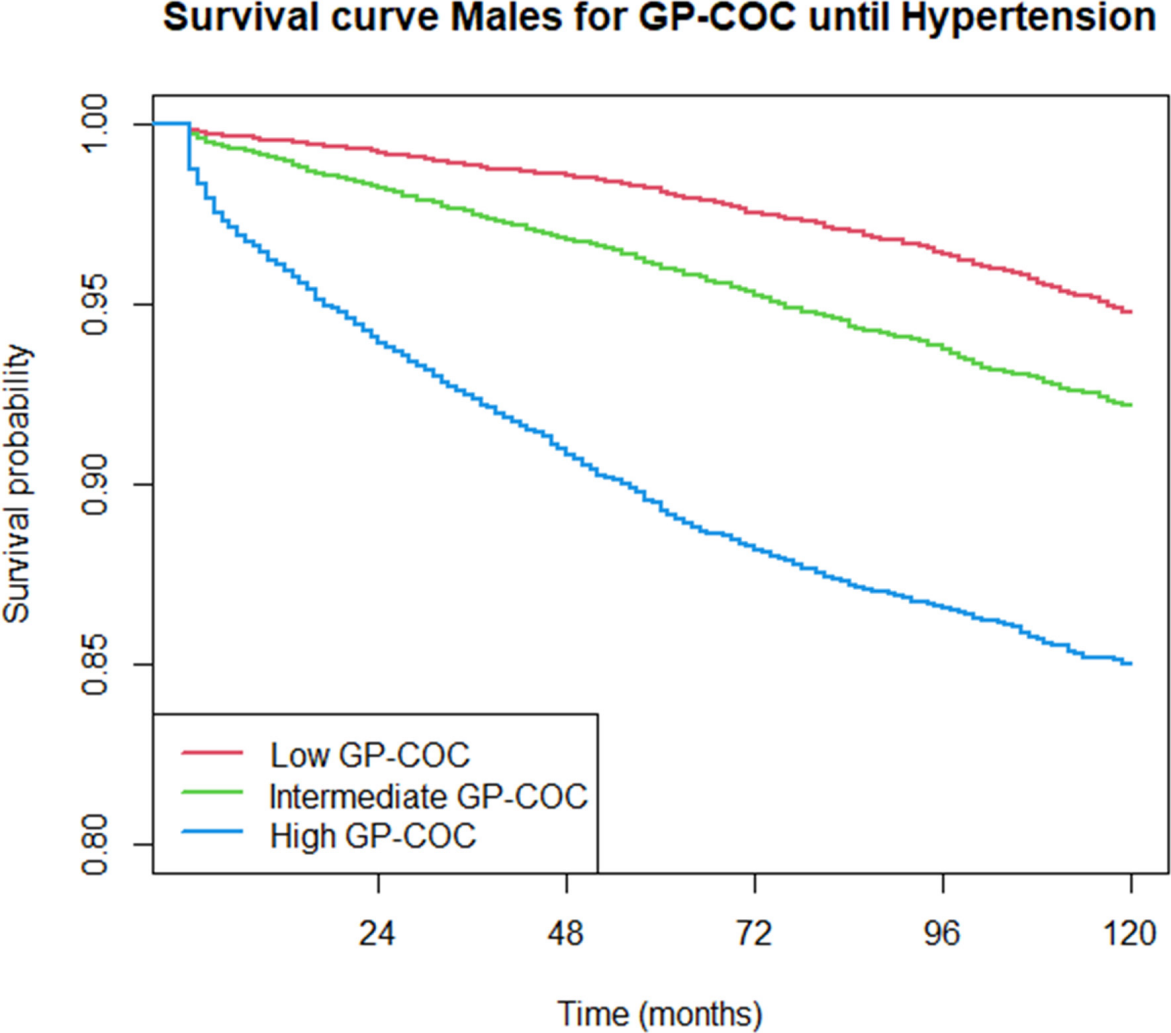
Kaplan-Meier survival curves until hypertension detection per continuity of care level: males, GP-COC. COC, continuity of care; GP, general practitioner.

Over the 10-year follow-up time consisting of 754 144.7 person-years, a total of 7104 hypertension diagnoses were recorded between 2013 and 2022 (incidence rate 9.42 per 1000 person-years, 95% CI 9.20 to 9.64). The incidence rates were comparable between GP-COC and team-COC (online supplemental table S3.3). Figures2^4^ show descriptive Kaplan-Meier survival curves until hypertension detection per level GP-COC and team-COC; additional descriptives are shown in online supplemental table S3.4. Table 2 shows the differences in time to hypertension detection (RMST) across various levels of COC.

**Figure 3:**
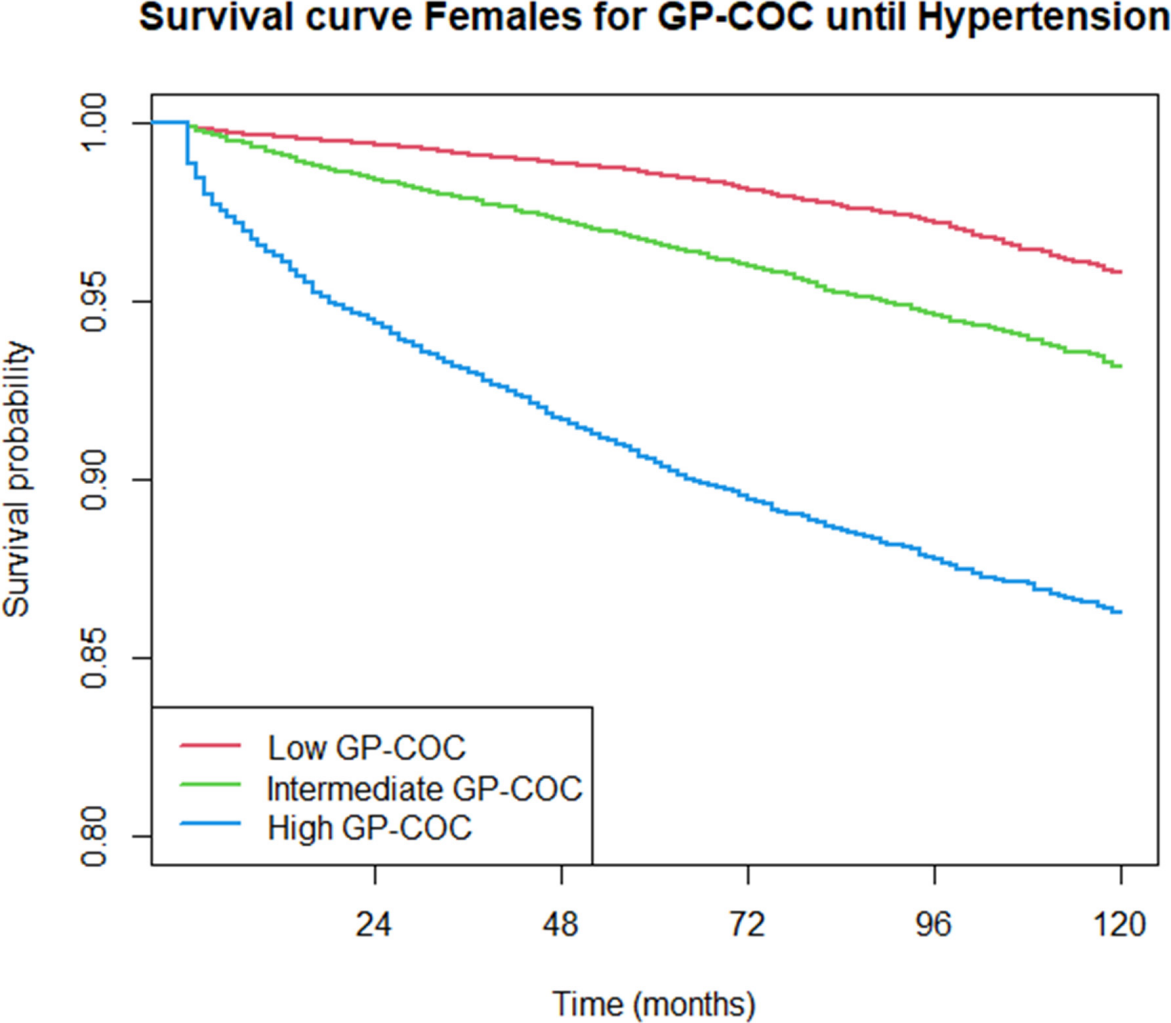
Kaplan-Meier survival curves until hypertension detection per continuity of care level: females, GP-COC. COC, continuity of care; GP, general practitioner.

**Figure 4:**
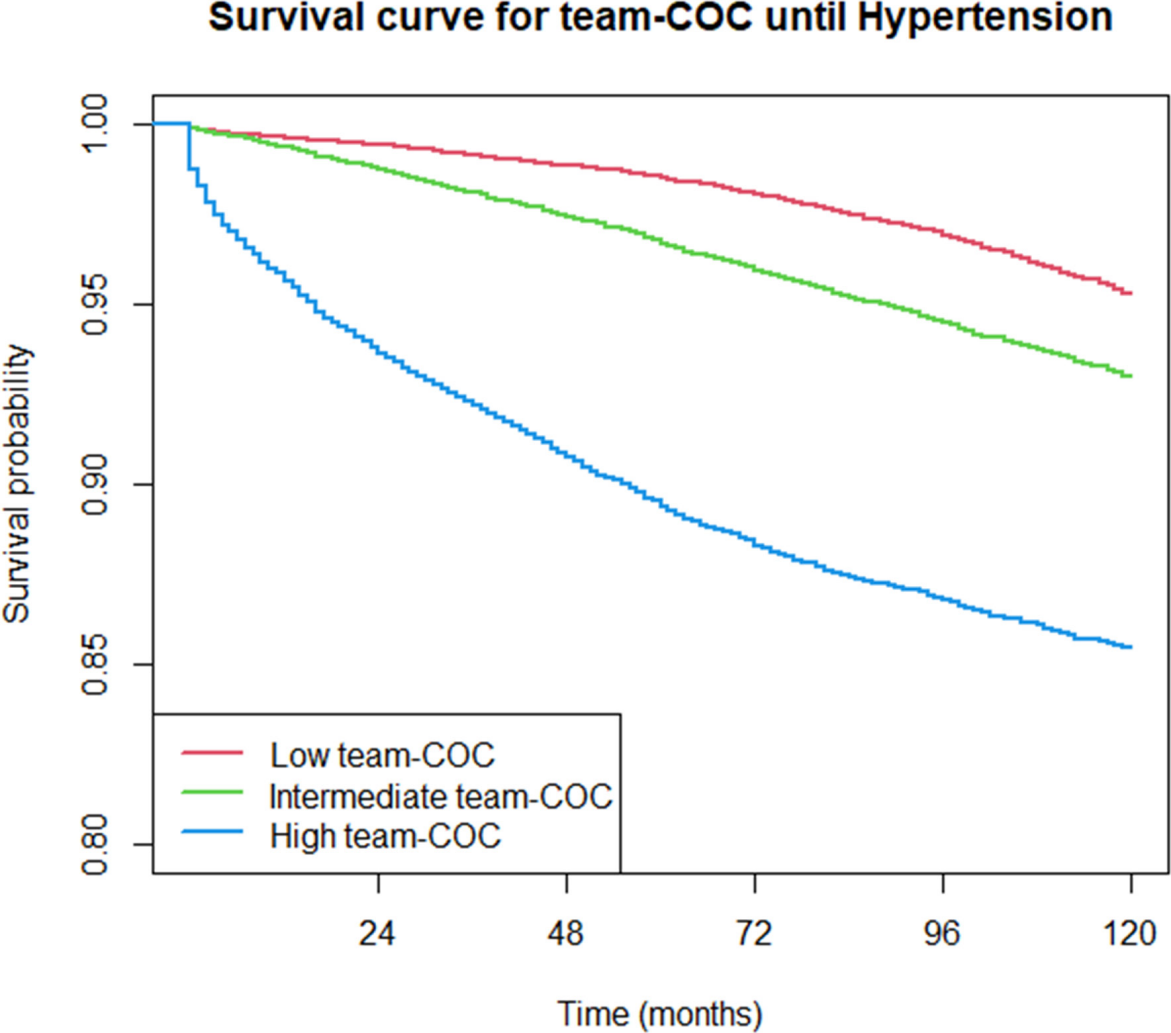
Kaplan-Meier survival curves until hypertension detection per continuity of care level: team-COC. COC, continuity of care.

**Table 2:** Differences in restricted mean survival time (RMST) within 10 years per continuity of care (COC) level

	RMST difference, months (95% CI) per continuity of care level (HHI)					
	Low vs intermediate COC		Intermediate vs high COC		Low vs high COC	
GP-COC	2.1	1.8 to 2.3	5.7	5.3 to 6.1	7.8	7.4 to 8.1
Male	2.1	1.7 to 2.5	5.9	5.3 to 6.5	8.0	7.5 to 8.5
Female	2.1	1.8 to 2.4	5.5	5.0 to 6.0	7.5	7.1 to 8.0
Team-COC	1.9	1.6 to 2.1	6.4	6.0 to 6.8	8.3	7.9 to 8.6

GP, general practitioner; HHI, Herfindahl-Hirschman Index.

For both GP-COC and team-COC, there was a dose–response relationship between COC level and time to hypertension detection. When following up the patients for 10 years, patients receiving high team-COC (RMST 109.7 months, 95% CI 109.4 to 110.0) have hypertension detected on average 8.3 months (95% CI 7.9 to 8.6) earlier compared with low team-COC (RMST 118.0 months, 95% CI 117.8 to 118.1). When compared with low GP-COC, patients receiving intermediate or high GP-COC had, respectively, 1.9 (95% CI 1.7 to 2.1) to 4.9 (95% CI 4.4 to 5.4) higher rates of hypertension detection over time (p<0.001), meaning that hypertension was detected more rapidly for patients receiving intermediate or high GP-COC compared with patients receiving low GP-COC. Patients receiving intermediate or high team-COC had, respectively, a 2.3 (95% CI 2.2 to 2.5) to 7.3 (95% CI 6.8 to 7.8) higher HRs of hypertension detection at any point in time (p<0.001) (table 3).

**Table 3:** HRs per continuity of care level until hypertension detection

	Continuity of care (HHI)					
	Intermediate COC			High COC		
	HR	95% CI	P value	HR	95% CI	P value
GP-COC†	2.1	2.0 to 2.3	<0.001	5.6	5.2 to 6.0	<0.001
Male	1.9	1.7 to 2.1	<0.001	4.4	4.0 to 5.0	<0.001
Female	2.1	1.9 to 2.3	<0.001	4.9	4.4 to 5.4	<0.001
Team-COC	1.9	1.8 to 2.1	<0.001	5.8	5.4 to 6.2	<0.001
Adjusted‡	2.3	2.2 to 2.5	<0.001	7.3	6.8 to 7.8	<0.001

* Reference: low level of COC.

† Stratified data are adjusted for confounding by age and by the average contact frequency of the patients during the follow-up period. The number of chronic diseases, the socioeconomic status, the percentage of citizens with a non-western migration background in the postal code area and the presence of GP trainees showed no significant interference.

‡ Data are adjusted for confounding by the average contact frequency of the patients during the follow-up period. Age, sex, the number of chronic diseases, the socioeconomic status, the percentage of citizens with a non-western migration background in the postal code area and the presence of a GP trainees showed no significant interference.

COC, continuity of care; GP, general practitioner; HHI, Herfindahl-Hirschman Index.

## Discussion

### Principal findings

Our study using routine care data and prolonged follow-up shows that both GP-COC and team-COC are similarly and dose-dependently associated with an increased HR of detecting hypertension in adults without preregistered cardiovascular conditions or risk factors. When compared with low COC, high COC was associated with, on average, 8 months earlier hypertension detection.

### Strengths and limitations

To our knowledge, this study is the first to calculate team-COC using a well-established continuity measure, the HHI. Acknowledging the gradual shift from personal to more team-based continuity, we present both GP-COC and team-COC, making it possible to compare these two different proxies of COC.

Also, this study gives weight to the impact of COC on preventive care. While earlier studies have indicated that higher COC may enhance the uptake of preventive care, the supporting evidence is relatively weak, largely due to methodological heterogeneity, limited sample sizes and variable data quality.^3039 40 70^,^73^ Our study adds to this by providing robust quantitative evidence on the association between greater COC and earlier hypertension detection in general practice.

Another strength of this study is the large sample size combined with the 10-year observation period, allowing COC monitoring over a longer term and across different disease episodes. This approach made an appropriate time-dependent analysis feasible and helped to prevent time-dependent bias and temporal ambiguity between COC and the detection of hypertension.^49 74 75^ Moreover, as data were enriched with publicly available environmental and lifestyle indicators, the study provides a comprehensive assessment of patient context. As the COVID-19 pandemic was part of the prolonged follow-up period, with potentially decreased COC and less (preventive care and vis-à-vis) consultations, we conducted a sub-analysis restricted to the pre-COVID period (until 1 January 2020). This sub-analysis revealed even higher HRs of hypertension detection for both GP-COC and team-COC compared with the prolonged follow-up period. These findings underscore the robustness of our main results (online supplemental table S3.5).

Some limitations must be acknowledged. First, there is no consensus in the current literature on how to measure COC.^62 63^ Various measures, including the Usual Provider Continuity Index (UPC) and the Bice-Boxerman Continuity of Care Index (mathematically similar to the HHI), are used interchangeably. Previous studies have shown high correlations between these measures.^76^ As there is no gold standard for measuring COC, the choice of measure can be guided by the specific intent and focus of the analysis.^76 77^ As the HHI incorporates both the frequency and dispersion of patient contacts across healthcare providers, it was considered the most appropriate measure to reflect and compare GP-COC and team-COC in this study. In contrast, the UPC is a density measure that does not account for the number of providers or the distribution of visits. In a sub-analysis using the UPC, a similar dose-dependent association between team-COC and hypertension detection was observed, although with lower HRs, corresponding to an average of 6 months earlier hypertension detection (see online supplemental tables S3.6 and S3.7). Regardless of whether the UPC is an ideal measure for team-based COC, the consistent dose-dependent findings support the robustness of our main results.

Second, routine care data have intrinsic limitations. Previous studies have shown that the validity of routine care data based on ICPC codes can be suboptimal due to variability in documentation practices between institutions and healthcare providers.^78^ Selective reporting also presents a challenge. Diagnoses may not always be recorded correctly according to guidelines, or an ICPC code may be registered as testing was performed, even if the diagnosis was ultimately ruled out.^79^ Under-reporting or over-reporting by GPs may result in a respective underestimation or overestimation of the actual effects in the analysis. By adding a frailty model on the 48 different general practices, we aimed to adjust for clustering at the practice level (eg, ICPC validation and rurality). Although the 10-year prevalence of hypertension in our dataset (6.7%) aligns with national prevalence,^80^ the generalisability of our findings beyond healthcare systems similar to the Dutch primary care context remains uncertain. The observed effect sizes may be larger than in less integrated systems, though the direction of the association is expected to be consistent.

### Comparison with existing literature

Previous studies have operationalised team-COC by comparing collaborating physicians to non-collaborating physicians.^5381^,^83^ However, this approach does not capture the broader practice team-COC that includes nurses and assistants. Studies which did incorporate the multidisciplinary nature of team-COC used a survey instead of an established measure as a proxy for team-COC.^84 85^

Regarding the association between COC and CVR detection, Koopman and colleagues investigated the impact of having a usual care provider on the recognition of CVRs by comparing survey data with laboratory findings.^86^ Although their study is methodologically different from our study, their findings align with our results: higher COC (having a usual place and provider vs no usual source of care) was associated with a lower likelihood of undiagnosed diabetes and hypertension (OR 0.30; 95% CI 0.10 to 0.95). A Chileshe cross-sectional study found a positive association between COC (measured by the question: ‘do you know the name of your family doctor?’) with high cardiovascular risk (OR 2.98, 95% CI 2.13 to 4.17), but was not associated with higher odds of being aware of their hypertension diagnosis, receiving pharmacological treatment or better levels of BP control.^87^ Another more recent study found no significant association between COC and the rate of, or time to, hypertension detection.^88^ However, they defined the time to hypertension detection as the registration of the diagnosis after reading the second reading of the BP, which is incomparable with our methodology.

Our study did not identify patient or practice characteristics associated with (dis)continuity of care or delayed detection as other studies did.^37 89^ Although other studies have emphasised the importance of promoting COC in older adults or patients with multiple chronic conditions, our study clearly demonstrates that fostering COC has a significant impact on the timely detection of hypertension in younger adults (median age 35, IQR 27–48 years) without a relevant medical history.

### Possible explanations and implications for researchers, clinicians and policymakers

Since hypertension is mostly diagnosed via opportunistic screening, fostering COC in general practice contributes to effective and efficient implementation of timely detection. Our findings align with expectations and the limited literature available. The magnitude of the effect underscores the potential value for clinicians and policymakers in making COC a tangible lever to enhance preventive cardiovascular care, especially in the early detection phase of disease prevention. Hence, we recommend prioritising the optimisation and protection of COC in the cardiovascular risk management infrastructure, for example, through organisational changes (eg, pro-active allocation of patients to named GPs, work in pairs, one GP per disease episode^90 91^) and patient education on the importance of COC and the function of practice healthcare workers.^37 64 90^ At the same time, it remains important to look critically at the cardiovascular risk management infrastructure itself and identify areas for improvement (eg, proactive case finding strategies and digital decision-support tools), enabling primary care to optimally detect and treat hypertension.

### Conclusions

In conclusion, a dose-dependent association was found between COC and timely hypertension detection in general practice. These findings highlight the clinical relevance of fostering COC, even in relatively young or low-risk patients. We advocate for strengthening COC within the framework of preventive cardiovascular care in general practice.

## Supplementary material

10.1136/bmjopen-2025-113374 online supplemental file 1

## Data Availability

Data may be obtained from a third party and are not publicly available.
