# Supplementary material for: Association between continuity of care and detection of hypertension in Dutch general practice: a 10-year cohort study
Source: BMJ Open. 2026 Mar 23;16(3):e113374. doi: 10.1136/bmjopen-2025-113374 (PMC13034284; doi:10.1136/bmjopen-2025-113374)
Supplement: online supplemental file 1 [file bmjopen-16-3-s001.docx]

# Supplementary data

## S1. International Classification of Primary Care-codes

**Table S1.** International Classification of Primary Care-codes included in study

|  | | | ICPC 11 |
| --- | --- | --- | --- |
| Cardiovascular risk factors | | |  |
|  | Hypertension | |  |
|  |  | Hypertension uncomplicated | K86 |
|  |  | Hypertension complicated | K87 |
|  | Hypercholesterolemia | |  |
|  |  | Lipid disorder | T93 |
|  |  | Hypercholesterolemia | T93.01 |
|  |  | Hypertriglyceridemia | T93.02 |
|  |  | Mixed hyperlipidaemia | T93.03 |
|  | Diabetes Mellitus (only included if T90.01 (T1DM) was not recorded) | | T90 |
|  |  | Type 2 Diabetes Mellitus | T90.02 |
| Chronic diseases | | |  |
|  |  | Limited function/disability NOS | A28 |
|  |  | Malignancy NOS | A79 |
|  |  | Congenital anomaly OS/multiple | A90 |
|  |  | Limited function/disability blood/lymphatic system | B28 |
|  |  | Hodgkin's disease/lymphoma | B72 |
|  |  | Leukaemia | B73 |
|  |  | Malignant neoplasm blood other | B74 |
|  |  | Hereditary haemolytic anaemia | B78 |
|  |  | Congenital anomaly blood/lymph other | B79 |
|  |  | Purpura/coagulation defect | B83 |
|  |  | HIV-infection/AIDS | B90 |
|  |  | Limited function/disability gastrointestinal tract | D28 |
|  |  | Malignant neoplasm stomach | D74 |
|  |  | Malignant neoplasm colon/rectum | D75 |
|  |  | Malignant neoplasm pancreas | D76 |
|  |  | Malignant neoplasm digestive other/NOS | D77 |
|  |  | Congenital anomaly digestive system | D81 |
|  |  | Diverticular disease | D92 |
|  |  | Chronic enteritis/ulcerative colitis | D94 |
|  |  | Liver disease NOS | D97 |
|  |  | Limited function/disability eyes/adnexa | F28 |
|  |  | Congenital anomaly eye other | F81 |
|  |  | Retinopathy | F83 |
|  |  | Macular degeneration | F84 |
|  |  | Refractive error | F91 |
|  |  | Glaucoma | F93 |
|  |  | Blindness | F94 |
|  |  | Limited function/disability ear | H28 |
|  |  | Congenital anomaly of ear | H80 |
|  |  | Otosclerosis | H83 |
|  |  | Presbycusis | H84 |
|  |  | Acoustic trauma | H85 |
|  |  | Deafness | H86 |
|  |  | Limited function/disability cardiovascular system | K28 |
|  |  | Congenital anomaly cardiovascular | K73 |
|  |  | Ischaemic heart disease w. angina | K74 |
|  |  | Other/Chronic Ischemic Heart Disease | K76 |
|  |  | Heart failure | K77 |
|  |  | Pulmonary heart disease | K82 |
|  |  | Hypertension uncomplicated | K86 |
|  |  | Hypertension complicated | K87 |
|  |  | Stroke/cerebrovascular accident | K90 |
|  |  | Atherosclerosis | K91 |
|  |  | Other Peripheral Arterial Diseases | K92 |
|  |  | Limited function/disability musculoskeletal system | L28 |
|  |  | Congenital anomaly musculoskeletal | L82 |
|  |  | Back syndrome w/o radiating pain | L84 |
|  |  | Acquired deformity of spine | L85 |
|  |  | Rheumatoid/seropositive arthritis | L88 |
|  |  | Osteoarthrosis of hip | L89 |
|  |  | Osteoarthrosis of knee | L90 |
|  |  | Osteoarthrosis other | L91 |
|  |  | Osteoporosis | L95 |
|  |  | Acquired deformity of limb | L98 |
|  |  | Limited function/disability (n) | N28 |
|  |  | Poliomyelitis | N70 |
|  |  | Malignant neoplasm nervous system | N74 |
|  |  | Congenital anomaly neurological | N85 |
|  |  | Multiple sclerosis | N86 |
|  |  | Parkinsonism | N87 |
|  |  | Epilepsy | N88 |
|  |  | Limited Function/Disability Due to Mental Illness | P28 |
|  |  | Dementia/Alzheimer’s disease | P70 |
|  |  | Schizophrenia | P72 |
|  |  | Personality disorder | P80 |
|  |  | Mental retardation | P85 |
|  |  | Limited Function/Disability of the Respiratory System | R28 |
|  |  | Malignant neoplasm bronchus/lung | R84 |
|  |  | Malignant neoplasm respiratory, other | R85 |
|  |  | Congenital anomaly respiratory | R89 |
|  |  | Congenital anomaly respiratory | R91 |
|  |  | Chronic obstructive pulmonary disease (COPD) | R95 |
|  |  | Asthma | R96 |
|  |  | Limited Function/Disability of the Skin/Subcutaneous Tissue | S28 |
|  |  | Malignant neoplasm of skin | S77 |
|  |  | Haemangioma/lymphangioma | S81 |
|  |  | Congenital skin anomaly other | S83 |
|  |  | Dermatitis/atopic eczema | S87 |
|  |  | Psoriasis | S91 |
|  |  | Limited Function/Disability of Endocrine Glands/Metabolism/Nutrition | T28 |
|  |  | Malignant neoplasm thyroid | T71 |
|  |  | Thyroglossal duct/cyst | T78 |
|  |  | Congenital anomaly endocrine/metabolic | T80 |
|  |  | Goitre | T81 |
|  |  | Hypothyroidism/myxoedema | T86 |
|  |  | Diabetes mellitus | T90 |
|  |  | Vitamin/nutritional deficiency | T92 |
|  |  | Lipid disorder | T93 |
|  |  | Limited function/disability urinary tract | U28 |
|  |  | Malignant neoplasm of kidney | U75 |
|  |  | Malignant neoplasm of bladder | U76 |
|  |  | Malignant neoplasm urinary other | U77 |
|  |  | Congenital anomaly urinary trac | U85 |
|  |  | Glomerulonephritis/nephrosis | U88 |
|  |  | Limited Function/Disability Due to Pregnancy | W28 |
|  |  | Malignant neoplasm relate to pregnancy | W72 |
|  |  | Congenital anomaly complicate pregnancy | W76 |
|  |  | Limited Function/Disability of Female Genital Organs | X28 |
|  |  | Other Symptoms/Complaints of Female Genital Organs | X75 |
|  |  | Malignant neoplasm breast female | X76 |
|  |  | Malignant neoplasm genital other | X77 |
|  |  | Congenital anomaly genital female | X83 |
|  |  | Fibrocystic disease breast | X88 |
|  |  | Limited Function/Disability of Male Genital Organs | Y28 |
|  |  | Malignant neoplasm prostate | Y77 |
|  |  | Malignant neoplasm male genital other | Y78 |
|  |  | Hypospadias | Y82 |
|  |  | Congenital genital anomaly other | Y84 |
|  |  | Social Functioning Disability | Z28 |
| Psychiatric disease | | |  |
|  |  | Schizophrenia | P72 |
|  |  | Affective psychosis | P73 |
|  |  | Anxiety disorder/anxiety state | P74 |
|  |  | Depressive disorder | P76 |
|  |  | Personality disorder | P80 |
| Excluded cardiovascular conditions/risk factors if registered < 2013/01/01 | | |  |
|  | Cardiovascular diseases | |  |
|  |  | Acute Myocardial Infarction | K75 |
|  |  | Other/Chronic Ischemic Heart Disease | K76 |
|  |  | Coronary Arteriosclerosis | K76.01 |
|  |  | Old Myocardial Infarction (> 4 weeks ago) | K76.02 |
|  |  | Angina pectoris | K74 |
|  |  | Unstable angina pectoris | K74.01 |
|  |  | Stable angina pectoris | K74.02 |
|  |  | Transient Cerebral Ischemia/  Transient Ischemic Attack (TIA) | K89 |
|  |  | Cerebral Infarction | K90.03 |
|  |  | Atherosclerosis | K91 |
|  |  | Other Peripheral Arterial Diseases | K92.01 |
|  |  | Aortic Aneurysm | K99.01 |
|  | Conditions and risk factors that (potentially) lead to an increased cardiovascular risk | |  |
|  |  | Nicotine dependence | P17 |
|  |  | Obesity | T82 |
|  |  | Essential hypertension without organ damage | K86 |
|  |  | Hypertension with organ damage / secondary hypertension | K87 |
|  |  | Elevated blood pressure | K85 |
|  |  | Lipid metabolism disorders | T93 |
|  |  | Hypercholesterolemia | T93.01 |
|  |  | Mixed hyperlipidemia | T93.03 |
|  |  | Family history of cardiovascular disease | A29.01 |
|  |  | Family history of hypercholesterolemia | A29.06 |
|  |  | Diabetes mellitus type 2 | T90.02 |
|  |  | Diabetes mellitus type 1 | T90.01 |
|  |  | Impaired glucose tolerance | A91.05 |
|  |  | Renal dysfunction / renal insufficiency | U99.01 |
|  |  | Other diseases of the urinary tract | U99 |
|  |  | Albuminuria | U98.03 |
|  |  | Intracerebral hemorrhage | K90.02 |
|  |  | Psoriatic arthritis | L99.13 |
|  |  | Chronic obstructive pulmonary disease (COPD) | R95 |
|  |  | Gout | T92 |
|  |  | Rheumatoid arthritis / related disorders | L88 |
|  |  | Rheumatoid arthritis | L88.01 |
|  |  | Ankylosing spondylitis | L88.02 |
|  |  | HIV infection (AIDS/ARC) | B90 |
|  |  | HIV positive without symptoms | B90.01 |
|  |  | AIDS/ARC | B90.02 |
|  |  | Ulcerative colitis / chronic (regional) enteritis | D94 |
|  |  | Ulcerative colitis | D94.01 |
|  |  | Crohn’s disease | D94.02 |
|  |  | Sleep apnea syndrome | P06.01 |
|  |  | Toxicosis / (pre-)eclampsia | W81 |
|  |  | Polycystic ovary syndrome (PCOS) | T99.06 |
|  |  | Gestational diabetes | W84.02 |
|  | Oncological diseases | |  |
|  |  | Malignant neoplasm unknown primary location | A79 |
|  |  | Lymphoma | B72 |
|  |  | Hodgkin's disease | B72.01 |
|  |  | Non-Hodgkin lymphoma | B72.02 |
|  |  | Leukaemia | B73 |
|  |  | Malignant neoplasm stomach | D74 |
|  |  | Malignant neoplasm colon/rectum | D75 |
|  |  | Malignant neoplasm pancreas | D76 |
|  |  | Malignant neoplasm other/unspecified | D77 |
|  |  | Malignant neoplasm oesophagus | D77.01 |
|  |  | Malignant neoplasm salivary glands | D77.02 |
|  |  | Malignancy of the lip/mouth/tongue | D77.03 |
|  |  | Malignant neoplasm liver/gallbladder/biliary tract | D77.04 |
|  |  | Malignant neoplasm musculoskeletal system | L71 |
|  |  | Musculoskeletal malignancy | L71.01 |
|  |  | Malignant neoplasm nervous system | N74 |
|  |  | Bronchial/lung malignancy | R84 |
|  |  | Other respiratory tract malignancy | R85 |
|  |  | Malignant neoplasm skin | S77 |
|  |  | Basal cell carcinoma | S77.01 |
|  |  | Squamous cell carcinoma | S77.02 |
|  |  | Malignant melanoma | S77.03 |
|  |  | Karposi’s sarcoma | S77.04 |
|  |  | Malignant neoplasm thyroid | T71 |
|  |  | Malignant neoplasm kidney | U75 |
|  |  | Malignant neoplasm bladder | U76 |
|  |  | Malignant neoplasm urinary tract, other | U77 |
|  |  | Malignant neoplasm cervix | X75 |
|  |  | Breast cancer, female | X76 |
|  |  | Breast adenocarcinoma, female | X76.01 |
|  |  | Other reproductive organ malignancy, female | X77 |
|  |  | Endometrial carcinoma | X77.01 |
|  |  | Ovarian malignancy | X77.02 |
|  |  | Prostate malignancy | Y77 |
|  |  | Other reproductive organ/breast malignancy, male | Y78 |
|  |  | Penile malignancy | Y78.01 |
|  |  | Testicular malignancy | Y78.02 |
|  |  | Breast malignancy, male | Y78.03 |

## S2. Additional Methodological Details for Lifestyle and Environmental factors

Since lifestyle factors such as tobacco use, alcohol consumption, overweight, and physical activity are underreported or reported late in general practice[1-4], and consequently in our available administrative routine care data, these variables were calculated per four-digit postal code using publicly available data derived from a model calculation by the National Institute for Public Health and the Environment (RIVM), based on data from the Health Monitor Adults and Elderly 2016, conducted by the Municipal Health Services (GGDs), Statistics Netherlands (CBS), and the RIVM[5]. The figures are estimated percentages for indicators related to health, social situations, and lifestyle at the neighbourhood, district, and municipality levels. This National Health Monitor provides annual information on the health, social situation, and lifestyle of a random selection from the Personal Records Database (Dutch abbreviation: BRP) aged 18 years and older[6].

Health Monitor Adults and Elderly

Data were collected by a mixed-model design, which is a combination of Computer Assisted Web Interviewing (CAWI, online respondents) and Computer Assisted Personal Interviewing (CAPI, face-to-face interviewing for non-respondents). Common topics included self-perceived health, chronic conditions, caregiving, height and weight, smoking, alcohol consumption, and physical activity. For modelling the estimates, participants of the Health Monitors were anonymously linked to CBS registry data within a secure environment. These registries contain background information on variables such as age, sex, ethnicity, household composition, educational level, income, housing type, and spatial location. A model using the XGBoost method, a machine learning algorithm often used for regression and classification tasks handling complex data relationships, was applied to relate health and lifestyle data to these background characteristics. Based on these relationships, it became possible to estimate the expected health and lifestyle outcomes for all adults. The resulting estimates were then averaged at the neighbourhood or district level.

Socioeconomic status

In addition, a socioeconomic status (SES-WOA) score based on household data concerning relative welfare (in deciles, based on a combination of spendable income and wealth), highest level of education and recent labour participation was publicly available published for all four-digit postal codes by Statistics Netherlands [7-9]. Ethnicity per four-digit postal code was separately extracted from Statistics Netherlands. This includes the percentage of the population who, or whose parents, were born in Africa, South America, Asia (excluding Indonesia and Japan) or Turkey in 2016.

Air pollution

Air pollution is also a well-established risk factor for cardiovascular diseases[10]. Both long-term and short-term exposure to particulate matter can cause severe health damage. Overall, exposure to particulate matter accounts for approximately 4% of the disease burden in the Netherlands, ranking among the top three leading risk factors for morbidity and mortality. It falls below smoking (13%) but is comparable in magnitude to overweight (5%) and physical inactivity (3-4%)[11]. At our request, the RIVM calculated the unweighted average particulate matter concentration (PM_2.5_ and PM_10_, µg/m³) for each four-digit postal code, which was subsequently provided for analysis[12].

The particulate matter (PM) concentrations were calculated using the monitoring tool of Central Instrument for Air Quality Monitoring (CIMLK, former NSL) from the RIVM. These calculations were performed for all address locations in the Netherlands, CIMLK assessment point locations, and rural areas without roads or buildings. The concentrations at these locations were interpolated into a nationwide grid file with a resolution of 25x25 or 10x10 meters, depending on the year and the size of the particulate matter. The RIVM, on behalf of the Dutch Ministry of Infrastructure and Water Management, created a map of these PM_2.5_ and PM_10_ concentrations for the Environmental Atlas.

ANHA-datamanagers matched the publicly available data at four-digit postal code level from the RIVM adjusted CBS/GGD Health Monitor 2016, the CBS-calculated SES-WOA 2016, and the RIVM average particulate matter concentrations (PM_2.5_ and PM_10_) for 2013, 2016, and 2022 with the four-digit postal codes of all patients included in the administrative routine care data from 2013 to 2022.

## S3. Supplementary results

**Table S3.1.** Number and percentage per profession and consultation setting

|  | Total number of consultations | | | | |
| --- | --- | --- | --- | --- | --- |
|  | GP’s practice | Home visits | Telephone | Email | Total |
| Usual GP | 1,378,532 (73.5) | 32,391 (1.7) | 431,462 (23.0) | 34,300 (1.8) | 1,876,685 (52.4) |
| Locum GP | 262,696 (78.6) | 3,716 (1.1) | 64,665 (19.3) | 3,139 (0.9) | 334,216 (9.3) |
| Other^a^ | 866,613 (63.2) | 14,184 (1.0) | 483,186 (35.3)^b^ | 6,636 (0.5)^b^ | 1,370,619 (38.3) |
| Total | 2,507,841 (70.0) | 50,291 (1.4) | 979,313 (27.3) | 44,075 (1.2) | 3,581,520 |
| Abbreviations: GP, General practitioner.  ^a^ These healthcare professionals are unspecified in the dataset and include among other professions: nurses for somatic diseases, nurses for psychiatric diseases, nurses for elderly care, locum nurses, assistants and locum assistants.  ^b^ These contacts were excluded in team-COC calculations via the Herfindahl-Hirschman Index. | | | | | |

| Table S3.2. Descriptive practice characteristics and behaviour/environmental factors | | | | | | | | | |
| --- | --- | --- | --- | --- | --- | --- | --- | --- | --- |
|  | | | | **Team-based personal Continuity of Care (HHI)** | | | | | **Total** |
|  |  |  |  | Low | Intermediate | | High | |  |
| Practice Characteristics | | | |  |  | |  | |  |
|  | N usual GPs (x, s) | | | 5.75 (2.32) | 5.27 (2.44) | | 5.08 (2.53) | | 5.36 (2.45) |
|  | N locum GPs (M, IQR) | | | 5 (2-7) | 4 (2-7) | | 4 (1-8) | | 4 (2-7) |
|  | N other providers (M, IQR) | | | 33 (25-49) | 33 (20-49) | | 30 (18-50) | | 32 (20-50) |
|  | Duration of Employment per usual GP, years (x, s) | | | 4.17 (1.06) | 4.51 (1.00) | | 4.65 (0.99) | | 4.44 (1.04) |
|  | N usual GPs working > 5years (M, IQR) | | | 2 (2-5) | 2 (2-5) | | 3 (2-4) | | 2 (2-5) |
|  | N patients per practice (M, IQR) | | | 4,216  (3,093-6,777) | 3,793  (2,841-6,6853) | | 3,504  (2,841-6,853) | | 4,064  (2,842-6,853) |
|  | N patients in practice with  GP trainees (n, %) | | | 28,509 (80.0) | 25,717 (72.9) | | 23,247 (64.9) | | 77,473 (72.6) |
|  | Enrolment period per patient^a^, months (M, IQR) | | | 144 (87-246) | 126 (74-226) | 108 (56-201) | | 126 (73-222) | |
|  |  | | < 5 years (n, %) | 3,243 (9.1) | 6,001 (17.0) | 9,461 (26.4) | | 18,705 (17.5) | |
|  |  | | 5-10 years (n, %) | 11,227 (31.5) | 10,444 (29.6) | 9,660 (27.0) | | 31,331 (29.3) | |
|  |  | | 10-15 years (n, %) | 6,604 (18.5) | 5,919 (16.8) | 5,649 (15.8) | | 18,172 (17.0) | |
|  |  | | 15-20 years (n, %) | 4,613 (12.9) | 4,435 (12.6) | 4,480 (12.5) | | 13,528 (12.7) | |
|  |  | | >20 years (n, %) | 8,961 (25.1) | 7,581 (21.5) | 5,797 (16.2) | | 22,339 (20.9) | |
|  |  | | Unknown | 998 (2.8) | 896 (2.5) | 786 (2.2) | | 2,680 (2.5) | |
| Neighbourhood data^b^ | | | |  |  | |  | |  |
|  | Health perception “good” or “excellent” (M, IQR) | | | 77 (73-79) | 78 (74-80) | | 78 (75-81) | | 78 (74-80) |
|  | Physical activity | | |  |  | |  | |  |
|  |  | Comply guidelines^c^ (M, IQR) | | 59 (53-64) | 60 (53-64) | | 60 (53-64) | | 59 (53-64) |
|  |  | Exercise weekly (M, IQR) | | 57 (53-59) | 58 (54-61) | | 58 (54-61) | | 58 (54-60) |
|  | Weight | | |  |  | |  | |  |
|  |  | Overweight^d^ (M, IQR) | | 39 (36-46) | 38 (35-43) | | 38 (34-43) | | 38 (35-43) |
|  |  | Obese^e^ (M, IQR) | | 11 (10-13) | 11 (9-12) | | 11 (9-12) | | 11 (9-12) |
|  | Tobacco use (x, s) | | | 25.82 (3.50) | 25.73 (3.74) | | 25.63 (3.96) | | 25.73 (3.74) |
|  | Alcohol use | | |  |  | |  | |  |
|  |  | Comply guidelines^f^ (M, IQR) | | 36 (31-42) | 35 (30-41) | | 34 (30-41) | | 35 (30-41) |
|  |  | Drinker^g^ (M, IQR) | | 82 (77-86) | 82 (78-86) | | 83 (79-86) | | 82 (78-46) |
|  |  | Heavy drinkers^h^ (M, IQR) | | 14 (11-17) | 15 (11-17) | | 15 (11-17) | | 15 (11-17) |
|  |  | Excessive drinkers^i^ (M, IQR) | | 10 (8-11) | 10 (8-12) | | 10 (8-12) | | 10 (8-12) |
|  | Air pollution^j^ | | |  |  | |  | |  |
|  |  | Coarse particles (M, IQR) | | 19.9 (19.3-21.0) | 20.2 (19.3-21.4) | | 20.2 (19.2-21.4) | | 20.2 (19.3-21.4) |
|  |  | Fine particles (M, IQR) | | 12 (11.4-12.8) | 12.2 (11.4-12.9) | | 12.2 (11.4-13.1) | | 12.1 (11.4-12.9) |
|  | Socioeconomic scores^k^ (x, s) | | | -0.38 (2.20) | -0.07 (2.09) | | 0.13 (2.03) | | -0.11 (2.12) |
|  |  | Low tertile (n, %) | | 13,236 (37.6) | 11,120 (32.0) | | 10,117 (28.9) | | 34,473 (32.8) |
|  |  | Intermediate tertile (n, %) | | 11,472 (32.5) | 11,352 (32.7) | | 11,381 (32.5) | | 34,205 (32.6) |
|  |  | High tertile (n, %) | | 10,537 (29.9) | 12,284 (35.3) | | 13,485 (38.5) | | 36,306 (34.6) |
|  | Non-western migration background^l^ (M, IQR) | | | 20.62  (17.49-39.35) | 20.62  (17.77-38.92) | | 20.62  (19.07-38.92) | | 20.62  (17.77-38.92) |
| Abbreviations: HHI, Herfindahl-Hirschman Index; GP, general practitioner; n, number; x, mean; s, standard deviation; M, median; IQR, interquartile range.  ^a^ Duration of enrolment within the same GPs practice until end of follow-up period.  ^b^ Averaged estimate percentage adults at four-digit postal code/neighbourhood level (2016) based on RIVM Health Monitor Adults (see Supplementary Methodology S2).  ^c^ Who engage in at least 150 minutes of moderate-intensity exercise per week, spread across multiple days (e.g., walking and cycling), and perform muscle- and bone-strengthening activities at least twice per week.  ^d^ Body Mass Index (BMI) ≥25 kg/m^2^.  ^e^ Body Mass Index (BMI) ≥30 kg/m^2^.  ^f^ Who drink no more than one glass of alcohol per day.  ^g^  Who drinks “sometimes”.  ^h^ Who drinks at least 4 glasses of alcohol (for women) or 6 glasses (for men) on a single day at least once per week.  ^i^ Who drink more than 14 glasses of alcohol per week (for women) or more than 21 glasses per week (for men).  ^j^ Mean concentration (atmospheric) particulate matter (μg/m^3^ ) of coarse particles (PM_10_) and Fine particles (PM_2.5_) in the postal code (2016).  ^k^ Socioeconomic status scores in 2016 neighbourhood compared to national average (0), based relative welfare, highest level of education and recent labour participation.  ^l^ Who, or whose parents, were born in Africa, South America, Asia (excluding Indonesia and Japan) or Turkey (2016). | | | | | | | | | |

**Table S3.3.** Incidence rates GP-based and Team-based Continuity of Care

|  | |  |  | Incidence rates | |
| --- | --- | --- | --- | --- | --- |
|  | | Person years | Events | Per 1000 person years | 95% CI |
| Overall | | 754,144.7 | 7,104 | 9.4 | 9.2-9.6 |
| GP-COC | | | | | |
|  | Low | 280,853.7 | 1,222 | 4.4 | 4.1-4.6 |
|  | Intermediate | 255,132.8 | 1,924 | 7.5 | 7.2-7.9 |
|  | High | 218,158.2 | 3,958 | 18.1 | 17.6-18.7 |
| Team-COC | | | | | |
|  | Low | 291,019.9 | 1,279 | 4.4 | 4.2-4.6 |
|  | Intermediate | 254,236.8 | 1,813 | 7.1 | 6.8-7.5 |
|  | High | 208,888.0 | 4,012 | 19.2 | 18.6-19.8 |

**Table S3.4.** Additional descriptives and statistics Kaplan-Meier Survival analysis

|  | | Total number of | | Log Rank (Mantel-Cox) | |
| --- | --- | --- | --- | --- | --- |
|  | | Patients | Hypertension diagnoses | Chi-square (*χ^2^*) test | *P* |
| GP-COC | |  |  |  |  |
| Male | | 43,180 | 3,318 | 969.734 | < 0.001 |
|  | Low | 13,463 | 552 |  |  |
|  | Intermediate | 14,280 | 882 |  |  |
|  | High | 15,437 | 1,884 |  |  |
| Female | | 63,501 | 3,785 | 1463.749 | < 0.001 |
|  | Low | 22,113 | 670 |  |  |
|  | Intermediate | 21,215 | 1,042 |  |  |
|  | High | 20,173 | 2,073 |  |  |
| Team-COC | | 106,683 | 7,103 | 2806.687 | < 0.001 |
|  | Low | 35,636 | 1,279 |  |  |
|  | Intermediate | 35,269 | 1,813 |  |  |
|  | High | 35,778 | 4,012 |  |  |

**Table S3.5.** Hazard ratios Continuity of Care until hypertension detection, before January 1^st^, 2020

|  | Continuity of Care (HHI) | | | | | |
| --- | --- | --- | --- | --- | --- | --- |
|  | Intermediate COC | | | High COC | | |
|  | HR^a^ | 95% CI | *P* | HR | 95% CI | *P* |
| GP-COC | 2.8 | 2.4-3.2 | < 0.001 | 5.9 | 5.2-6.7 | < 0.001 |
| Adjusted^b^ | 2.5 | 2.2-2.8 | < 0.001 | 4.6 | 4.1-5.2 | < 0.001 |
| Team-COC | 2.3 | 2.1-2.7 | < 0.001 | 7.9 | 7.0-8.4 | < 0.001 |
| Adjusted^b^ | 2.1 | 1.8-2.4 | < 0.001 | 6.3 | 5.5-7.1 | < 0.001 |
| Abbreviations: HR, Hazard Ratio; CI, Confidence Interval; COC, Continuity of Care; GP, General Practitioner.  ^a^ Reference: low level of COC.  ^b^ Data is adjusted for confounding by age. Sex, the number of chronic diseases, the socio-economic status, the percentage citizens with a non-western migration background in the postal code area and the presence of a GP trainees showed no significant interference. | | | | | | |

**Table S3.6** Hazard ratios per level Continuity of Care, calculated via the UPC-method, until hypertension detection

|  | | Continuity of Care (UPC) | | | | | |
| --- | --- | --- | --- | --- | --- | --- | --- |
|  | | Intermediate COC | | | High COC | | |
|  | | HR^a^ | 95% CI | *P* | HR | 95% CI | *P* |
| Team-COC | | 1.8 | 1.7-1.9 | < 0.001 | 4.0 | 3.8-4.3 | < 0.001 |
|  | Male^b^ | 1.6 | 1.4-1.8 | < 0.001 | 3.2 | 2.9-3.5 | < 0.001 |
|  | Female^b^ | 1.8 | 1.6-1.9 | < 0.001 | 3.4 | 3.1-3.7 | < 0.001 |
| Effect sizes are relative to low COC. The Usual Provider of Care Index (UPC) is calculated based on the fraction of contacts with a particular GP, $\boldsymbol{UPC= max}\left( \frac{\boldsymbol{n}_{\boldsymbol{i}}}{\boldsymbol{n}} \right)$, where n is the total number of contacts with any provider and n_i_ is the number of contacts with provider most seen. Abbreviations: COC, Continuity of Care; HR, Hazard Ratio; CI, Confidence Interval; GP, General Practitioner.  ^a^ Reference: low level of COC.  ^b^ Stratified data is adjusted for confounding by age, the number of chronic diseases and by the average contact frequency of the patients during the follow-up period. The socio-economic status, the percentage citizens with a non-western migration background in the postal code area and the presence of GP trainees showed no significant interference. | | | | | | | |

**Table S3.7** Differences in restricted mean survival time (RMST) within ten years per Continuity of Care level, calculated via the Usual Provider of Care Index (UPC)

|  | | RMST difference, months (95% CI) per Continuity of Care level (UPC) | | | | | |
| --- | --- | --- | --- | --- | --- | --- | --- |
|  | | Low vs. Intermediate COC | | Intermediate vs. High COC | | Low vs High COC | |
| Team-COC | | 1.9 | 1.6-2.1 | 4.4 | 4.0-4.7 | 6.2 | 5.9-6.6 |
|  | Male | 1.8 | 1.4-2.2 | 4.6 | 4.0-5.1 | 6.4 | 5.9-6.9 |
|  | Female | 1.9 | 1.6-2.2 | 4.2 | 3.7-4.6 | 6.0 | 5.6-6.5 |
| The Usual Provider of Care Index (UPC) is calculated based on the fraction of contacts with a particular GP, $\boldsymbol{UPC= max}\left( \frac{\boldsymbol{n}_{\boldsymbol{i}}}{\boldsymbol{n}} \right)$, where n is the total number of contacts with any provider and n_i_ is the number of contacts with provider *i*. Abbreviations: UPC, Usual Provider of Care; COC, Continuity of Care; RMST, Restricted Mean Survival Time; CI, Confidence Interval. | | | | | | | |

**References supplementary data**

1. Willemse, E., et al., *Stoppen‑met‑rokenzorg in de huisartsenpraktijk: een verkenning naar de stand van zaken.* Utrecht: Trimbos‑instituut, 2021.

2. Leemrijse, C., et al., *Monitor (vroeg)signalering alcoholproblematiek: Eerste meting in de eerstelijns- en tweedelijnszorg*. 2024, Nivel.

3. Leemrijse, C., L. Ramerman, and R. Verheij, *Alcoholproblematiek in de huisartsenzorg: Mogelijkheden voor vroegsignalering?* 2024, Nivel.

4. Kloek, C.J., et al., *Dutch General Practitioners' weight management policy for overweight and obese patients.* BMC Obes, 2014. **1**: p. 2.

5. Municipal Health Services (GGD), Statistics Netherlands (CBS), and National Institute for Public Health and the Environment (RIVM), *Health Monitor Adults and Elderly 2012/2016/2020/2022*, Adjusted based on the SMAP methodology by the RIVM, Editor. 2023, National Institute for Public Health and the Environment (RIVM): <https://statline.rivm.nl/#/RIVM/nl/dataset/50120NED/table?dl=B14A3>.

6. Statistics Netherlands (CBS). *Health Survey as of 2014*. [cited 2024 December 17]; Available from: <https://www.cbs.nl/en-gb/our-services/methods/surveys/brief-survey-description/health-survey-as-of-2014>.

7. Statistics Netherlands (CBS). *SES-WOA scores per wijk en buurt*. [cited 2024 December 9]; Available from: <https://www.cbs.nl/nl-nl/onze-diensten/methoden/onderzoeksomschrijvingen/korte-onderzoeksomschrijvingen/ses-woa-scores-per-wijk-en-buurt>.

8. Statistics Netherlands (CBS), *Sociaal-economische status (SES-WOA) per viercijferige postcode op 1 januari 2014 - 1 januari 2019; exclusief studentenhuishoudens*. 2022: <https://www.cbs.nl/nl-nl/maatwerk/2022/34/sociaal-economische-status-per-postcode-2014-2019>.

9. Arts, K., et al., *Berekenwijze Sociaal Economische Status scores*, in *PR001119*. 2021, Statistics Netherlands (CBS),: <https://www.cbs.nl/nl-nl/maatwerk/2021/45/berekenwijze-ses-score-per-wijk-buurt>.

10. U.S. Environmental Protection Agency, *Integrated Science Assessment for Particulate Matter*. 2009: Office of Research and Development, Research Triangle Park, North Carolina.

11. Gezondheidsraad, *Gezondheidseffecten luchtverontreiniging. Achtergronddocument bij het advies Gezondheidswinst door schonere lucht*. 2018, Gezondheidsraad: <www.gezondheidsraad.nl/documenten/adviezen/2018/01/23/gezondheidswinst-door-schonere-lucht>.

12. Atlas Leefomgeving (ALO). *Fijnstof 2013/2016/2022 (pm10, pm2.5)*. Available from: <www.atlasleefomgeving.nl>.
