# Supplementary material for: Association between continuity of care and detection of hypertension in Dutch general practice: a 10-year cohort study
Source: BMJ Open. 2026 Mar 23;16(3):e113374. doi: 10.1136/bmjopen-2025-113374 (PMC13034284; doi:10.1136/bmjopen-2025-113374)
Supplement: Supplementary file 2 [file bmjopen-2025-113374.reviewer_comments.pdf]

PEER REVIEW HISTORY

BMJ Open publishes all reviews undertaken for accepted manuscripts. Reviewers are asked to complete a checklist review form and are provided with free text boxes to elaborate on their assessment. These free text comments are reproduced below.

ARTICLE DETAILS

Title (Provisional)

The association between continuity of care and detection of hypertension in Dutch general practice: a 10-year cohort study

Authors

van der Velde, Nick Ryan; te Winkel, Marije T; Kanning, Jos P; Lissenberg-Witte, Birgit I; Harskamp, Ralf; Maarsingh, Otto R

VERSION 1 - REVIEW

**Reviewer**	**1**
**Name**	**Pereira Gray, Denis**
**Affiliation**	**St Leonard's Research Practice**
**Date**	**23-Dec-2025**
**COI**	**None**

Editor BMJ Open

22 December 2025

Thank you for inviting me to review this manuscript. I know of some of the authors and have corresponded with one of them but have no professional or financial connections with any of them. One of my articles has been cited. I believe I have no conflicts of interest in reviewing this submission.

Van der Velde N et al. The association between continuity of care and early hypertension detection in general practice: a ten-year cohort study

STRENGTHS

• This article has many strengths.

• This Department at Amsterdam has a strong academic record in research on continuity of care.

• The authors report on a good number of patients (>100K).

• It is a strength that they studied their data over ten years.

- Hypertension is a common and important medical condition and, as described, generates many consultations in general practice.
- It detection is the responsibility of general practice not hospitals and it is a condition mostly often treated in general practice in Europe.
- The diagnosis of hypertension is important as it is associated with some serious complications, like strokes.
- Whilst the treatment of hypertension is classified as the management of chronic disease it is simultaneously, as the authors rightly state, an example of the role of general practice providing an important form of preventive medicine.
- Treatment of hypertension to control it is available and effective.
- A strength is the authors reporting the use of the HH index to measure team continuity in general practice as well as GP continuity of care.
- The finding of a significant association between continuity and detection is important.
- A growing number of recent studies of continuity of care have reported a dose-response relationship for the continuity. This article adds to them by finding a dose- response relationship for both GP and primary care team continuity.
- The data gathered include information about some important social determinants of illness which may be valuable in future research.

LIMITATIONS

- In the title the word "early" is used in relation to detection and the authors make their point that patients who receive continuity of care had hypertension detected earlier than those in the low continuity (control) group. However, in this context the word 'early' is ambiguous as it does not mean that the patients detected were necessarily detected early in the course of their condition as they might have had the condition for a long time before the detection.
- The term hazard ratio is well established in the literature, but there is a dissonance for readers in English in describing the early diagnosis of hypertension as a "hazard". In fact early diagnosis is good clinical practice and the clinical hazard is among those not detected.
- The authors are exploring GP versus team continuity and are working at the front end of current interest in general practice. For their team calculations they have included the GPs as well. This is reasonable as the team obviously includes the GPs. However, it is also a form of double counting, as the GP continuity is reported separately and then again within the team continuity. The authors might consider reporting the continuity of the non GP -members of the team to add understanding.

RECOMMENDATION

This is an important article with some original findings. In its current form it has some limitations as set out and the authors should be given the opportunity to address these.

**Reviewer**	**2**
**Name**	**Sicari, Rosa**
**Affiliation**	**National Research Council, Institute of Clinical Physiology-Pisa**
**Date**	**14-Jan-2026**
**COI**	**None**

This is a well designed study demonstrating that increased continuity of care allows early hypertension detection, which may contribute to better cardiovascular outcomes. Authors analyze the time to hypertension detection across various levels of continuity of care. Moreover, continuity of care was calculated separately for GPs and for the entire general practice team. The study demonstrates that both GP and team continuity of care are dose-dependently associated with increased hazards and earlier detection of hypertension in adults without preregistered cardiovascular conditions. The study is well designed, the methodology is sound and conclusions are supported by results. Authors, on the basis of their findings recommend continuity of care as a main tool to prevention. It may seem a platitude but they demonstrate how a different modulation of continuity of care has a different clinical impact. There are a few issues to be addressed:

1. The model can be applied to the Netherland health system (and the city of Amsterdam in particular). How would Authors translate these findings to other systems? The organizations of GPs and other specialized teams seem to be crucial.

2. The Herfindahl-Hirschman Index is robust enough to detect the different levels to care but it does not include adherence to life-style changes. Please discuss

3. In the discussion there is too much emphasis on this being the first study using this methodology. It would be better to tone down the conclusions and focus more on how the health system should adhere to a close care of these patients in order to detect hypertension and treat them accordingly.

VERSION 1 - AUTHOR RESPONSE

Reviewer: 1
Prof. Denis Pereira Gray, St Leonard's Research Practice, University of Exeter
Comments to the Author:
Editor BMJ Open
22 December 2025
Thank you for inviting me to review this manuscript. I know of some of the authors and

have corresponded with one of them but have no professional or financial connections with any of them. One of my articles has been cited. I believe I have no conflicts of interest in reviewing this submission.

STRENGTHS

- **This article has many strengths.**
- **This Department at Amsterdam has a strong academic record in research on continuity of care.**
- **The authors report on a good number of patients (>100K).**
- **It is a strength that they studied their data over ten years.**
- **Hypertension is a common and important medical condition and, as described, generates many consultations in general practice.**
- **It detection is the responsibility of general practice not hospitals and it is a condition mostly often treated in general practice in Europe.**
- **The diagnosis of hypertension is important as it is associated with some serious complications, like strokes.**
- **Whilst the treatment of hypertension is classified as the management of chronic disease it is simultaneously, as the authors rightly state, an example of the role of general practice providing an important form of preventive medicine.**
- **Treatment of hypertension to control it is available and effective.**
- **A strength is the authors reporting the use of the HH index to measure team continuity in general practice as well as GP continuity of care.**
- **The finding of a significant association between continuity and detection is important.**
- **A growing number of recent studies of continuity of care have reported a dose-response relationship for the continuity. This article adds to them by finding a dose-response relationship for both GP and primary care team continuity.**
- **The data gathered include information about some important social determinants of illness which may be valuable in future research.**

We would like to thank the reviewer for these positive and encouraging comments.

LIMITATIONS

- **In the title the word "early" is used in relation to detection and the authors make their point that patients who receive continuity of care had hypertension detected earlier than those in the low continuity (control) group. However, in this context the word 'early' is ambiguous as it does not mean that the patients detected were necessarily detected early in the course of their condition as they might have had the condition for a long time before the detection.**

We agree that the word 'early' is ambiguous in this context. We removed 'early detection' from the title and changed it into:

"The association between continuity of care and detection of hypertension in Dutch general practice: a 10-year cohort study"

Also, we changed 'early detection' into 'timely detection' in the whole manuscript (page 2 line 32; page 5, line 111; page 15, line 333 and 339; page 16, line 352).

• The term hazard ratio is well established in the literature, but there is a dissonance for readers in English in describing the early diagnosis of hypertension as a "hazard". In fact early diagnosis is good clinical practice and the clinical hazard is among those not detected.

We agree that the word 'hazard' can create dissonance for English readers, as the connotation may be negative (i.e., hazard=danger) and the corresponding clinical effect positive (i.e., higher hazard of detection=early diagnosis=good clinical practice). To clarify this, we structurally changed the word 'hazard' to 'hazard ratio' (whole manuscript) and elaborated on the respective meaning in the Result paragraph (page 12, lines 242-246):

"When compared to low GP-COC, patients receiving intermediate or high GP-COC had respectively 1.9 (95%CI 1.7-2.1) to 4.9 (95%CI 4.4-5.4) higher rates of hypertension detection over time ($P < 0.001$), meaning that hypertension was detected more rapidly for patients receiving intermediate of high GP-COC compared to patients receiving low GP-COC."

• The authors are exploring GP versus team continuity and are working at the front end of current interest in general practice. For their team calculations they have included the GPs as well. This is reasonable as the team obviously includes the GPs. However, it is also a form of double counting, as the GP continuity is reported separately and then again within the team continuity. The authors might consider reporting the continuity of the non GP -members of the team to add understanding.

While we understand this wish from a clinical perspective, it falls outside the scope of our research. It was a deliberate choice to calculate both GP- and team-COC, particularly because team-COC is increasingly used and has rarely been studied. In addition, we consider both GP-COC and team-COC to be clinically more relevant than non-GP COC.

RECOMMENDATION
This is an important article with some original findings. In its current form it has some limitations as set out and the authors should be given the opportunity to address these.

We would like to thank the reviewer for his positive comments and we hope we have sufficiently addressed the respective limitations.

Reviewer: 2
Dr. Rosa Sicari, National Research Council
Comments to the Author:
This is a well designed study demonstrating that increased continuity of care allows early hypertension detection, which may contribute to better cardiovascular outcomes. Authors analyze the time to hypertension detection across various levels of continuity of care. Moreover, continuity of care was calculated separately for GPs and for the entire general practice team. The study demonstrates that both GP and team continuity of care are dose-dependently associated with increased hazards and earlier detection of hypertension in adults without preregistered cardiovascular conditions. The study is well designed, the methodology is sound and conclusions are supported by results. Authors, on the basis of their findings recommend continuity of care as a main tool to prevention. It may seem a platitude but they demonstrate how a different modulation of continuity of care has a different clinical impact.

We would like to thank the reviewer for the positive comments.

There are a few issues to be addressed:
1. The model can be applied to the Netherland health system (and the city of Amsterdam in particular). How would Authors translate these findings to other systems? The organizations of GPs and other specialized teams seem to be crucial.

We agree that the organizational characteristics of general practices may differ across countries and corresponding healthcare systems, which may affect COC and associated hypertension detection. For example, larger practices with more GPs may negatively affect the level of COC.[Forbes, *Brit J Gen Pract* 2020; Te Winkel, *Brit J Gen Pract* 2022]; Guthrie, *Fam Pract* 2002; Campbell, *Brit J Gen Pract* 2001]

We added this to the 'Strengths and limitations of this study' section (page 3, lines 69-70):

- The generalizability of our findings beyond health-care systems similar to Dutch primary care remains uncertain.

and we also elaborate on the generalizability of our findings in the Discussion paragraph (page 14, lines 306-310):

"Although the ten-year prevalence of hypertension in our dataset (6.7%) aligns with national prevalence(80), the generalizability of our findings beyond health-care systems similar to the Dutch primary care context remains uncertain. The observed effect sizes may be larger than in less integrated systems, though the direction of the association is expected to be consistent."

It is important, though, to emphasize that the robust scientific basis for previously identified beneficial effects of COC – e.g., fewer hospital admissions,[SR of Kao, *Am J Manag Care*

2019] reduced healthcare costs,[SR of Bazemore, *Ann Fam Med* 2023] and reduced mortality rates[SR of Pereira Gray, *BMJ Open* 2018] – is explicitly based on quantitatively measured COC (i.e., how often does a patient see his/her own GP and how does this compare to the total number of GP?). In other words, if practices actually succeed in significantly improving quantitative COC, it is likely that (previously proven) beneficial effects may occur – regardless of the practice characteristics. As this is the first study on COC and hypertension detection, it is important to remain modest and wait until – as with established beneficial effects of COC – the results have been replicated and duplicated.[Maarsingh, *Ann Fam Med* 2024] From an etiological perspective, though, the dose-dependent association between COC and hypertension detection seems promising.

2. The Herfindahl-Hirschman Index is robust enough to detect the different levels to care but it does not include adherence to life-style changes. Please discuss

COC indices, such as the Herfindahl-Hirschman Index, can quantitatively measure the degree of continuity of care. They are divided into indices that calculate COC on the duration of the relationship with the caregiver, the density of visits, the dispersion of caregivers, and the sequence of caregivers.[Jee, *Med Care Res Rev* 2006] Unfortunately, COC indices do not include adherence to life-style changes. Also, our dataset itself concerns routine care data and does not provide data on adherence to life-style changes.

3. In the discussion there is too much emphasis on this being the first study using this methodology. It would be better to tone down the conclusions and focus more on how the health system should adhere to a close care of these patients in order to detect hypertension and treat them accordingly.

We toned down the conclusion and removed "the first" twice (page 13, line 266 and lines 269-270):

"Also, this study gives weight to the impact of COC on preventive care."

and:

"Our study adds to this by providing robust quantitative evidence on the association between greater COC and earlier hypertension detection in general practice."

Also, we added a sentence that focuses on the importance of optimizing the cardiovascular risk management infrastructure itself (page 16, lines 346-349).

At the same time, it remains important to look critically at the cardiovascular risk management infrastructure itself and identify areas for improvement (e.g., proactive case finding strategies and digital decision-support tools), enabling primary care to optimally detect and treat hypertension.
